# New techniques versus standard mapping for sentinel lymph node biopsy in breast cancer: a systematic review and meta-analysis

**DOI:** 10.1007/s13304-023-01560-1

**Published:** 2023-06-16

**Authors:** Nicola Rocco, Nunzio Velotti, Martina Pontillo, Antonio Vitiello, Giovanna Berardi, Antonello Accurso, Stefania Masone, Mario Musella

**Affiliations:** 1 Department of Advanced Biomedical Sciences, University of Naples “Federico II”, Via S. Pansini, 5, 80131 Naples, Italy; 2 Department of Clinical Medicine and Surgery, University of Naples “Federico II”, Naples, Italy

**Keywords:** Sentinel lymph node biopsy, Breast cancer, Indocyanine green, Superparamagnetic iron oxide

## Abstract

New tracers for sentinel lymph node biopsy (SLNB), as indocyanine green (ICG), superparamagnetic iron oxide (SPIO) and micro bubbles, have been recently introduced in clinical practice showing promising but variable results. We reviewed the available evidence comparing these new techniques with the standard tracers to evaluate their safety. To identify all available studies, a systematic search was performed in all electronic databases. Data regarding sample size, mean number of SLN harvested for patient, number of metastatic SLN and SLN identification rate of all studies were extracted. No significant differences were found in terms of SLNs identification rates between SPIO, RI and BD but with a higher identification rate with the use of ICG. No significant differences were also found for the number of metastatic lymph nodes identified between SPIO, RI and BD and the mean number of SLNs identified between SPIO and ICG versus conventional tracers. A statistically significant differences in favor of ICG was reported for the comparison between ICG and conventional tracers for the number of metastatic lymph nodes identified. Our meta-analysis demonstrates that the use of both ICG and SPIO for the pre-operative mapping of sentinel lymph nodes in breast cancer treatment is adequately effective.

**Supplementary Information:**

The online version contains supplementary material available at 10.1007/s13304-023-01560-1.

## Introduction

Sentinel lymph node biopsy (SLNB) as an axillary staging procedure for breast cancer patients was introduced by Krag et al. and Giuliano et al. in the early nineties of the last Century [1, 2].

Since the pioneering experience with both radioisotope (RI) and Blue Dye (BD), SLNB has become the standard treatment for axillary staging in breast cancer patients that are node negative at pre-operative clinical examination and imaging [3].

Giuliano and Krag respectively proposed the SLN mapping with BD and RI. Both methods have been subsequently used as single technique or as a dual complementary procedure with reliable identification rates [4].

Some logistic issues due to the availability and disposal of radioisotopes, nuclear medicine facilities, costs and anaphylactic reactions to blue dye led to the search and development of new techniques. New tracers have been introduced in clinical practice as indocyanine green (ICG), superparamagnetic iron oxide (SPIO) and micro bubbles [5].

ICG is directly injected into the breast, subdermally in periareolar or retroareolar site. The SLNs are subsequently localized using a fluorescent imaging system [6–8].

SPIO is a magnetic tracer injected subcutaneously in the breast and identifying the SLNs within few minutes, with iron deposition in sinuses and macrophages. The SLNs are subsequently identified with a handheld magnetometer [9, 10].

These new techniques have been investigated in case series and prospective comparative studies, showing promising but variable results.

We reviewed the available evidence deriving from observational studies comparing new techniques for sentinel lymph node mapping with the standard tracers (RI, BD and dual technique) with the aim of reinforcing the safety of these techniques in terms of identification rates, number of SLNs identified and metastatic lymph nodes identification rates.

## Materials and methods

A protocol for this analysis was prospectively developed, with specific objectives, detailed criteria for study selection and evaluation of quality, identification of the outcomes and of the statistical methods. Ethical standards are not required for this review of literature.

### Literature search strategy

To identify all available studies, a systematic search was performed according to PRISMA (Preferred Reporting Items for Systematic reviews and Meta-Analyses) flowchart in all electronic databases (PubMed, Web of Science, Scopus, EMBASE). We used medical subject headings (MeSH) and free-text words using the following search terms in all possible combinations: sentinel, breast, blue, radioisotope, technetium, ICG, indocyanine, green, SPIO, magnetic. The last search was performed in March 2022.

According to PICO framework (Problem/Population, Intervention, Comparison and Outcome), study selection criteria was exactly defined. The search strategy was limited to articles written in English language.

### Studies selection and data extraction

Inclusion criteria regarded all studies reporting on breast cancer patients undergoing SLNB; only studies that compared the new methods of ICG and SPIO with the conventional tracers (radioisotope and blue dye) were included. Papers were eligible for inclusion if authors were able to extract data regarding the comparison between ICG or SPIO versus single or dual tracers conventional approach.

Studies not written in English, reviews, case report and papers regarding animal studies were excluded.

Two independent authors (NR, NV) analyzed each article and performed the data extraction independently. Duplicate studies were removed. Two other authors (AA, MP) further reviewed independently the eligibility of studies in abstract form and in full text by assessing if the inclusion criteria and outcome measures were met. In case of disagreement, a fourth investigator was consulted (AV). Discrepancies were resolved by consensus.

Data regarding sample size, mean number of SLN harvested for patient, number of metastatic SLN identified and SLN identification rate of all included studies were extracted.

### Statistical analysis

Dichotomous variables were pooled using the odds ratio (OR) with a 95% CI. The overall effect was tested using Z scores and significance was set at p < 0.05. Statistical analysis was realized with by using Comprehensive Meta-analysis [Version 2, Biostat, Englewood NJ (2005)]. Heterogeneity was investigated by the use of *I*^2^ statistic. For *I*^2^ of between 0 and 30%, heterogeneity was considered as probably not important, between 30 and 60% moderate, between 50 and 90% substantial, and between 75 and 100% considerable.

In order to be as conservative as possible, the random effect method was used for all analyses to take into account the variability among included studies.

### Risk of bias assessment

Publication bias was assessed by the Egger’s test and represented graphically by funnel plots for each outcome. Visual inspection of funnel plot asymmetry was performed to address for possible small-study effect, and Egger’s test was used to assess publication bias, over and above any subjective evaluation. [11] A p < 0.10 was considered statistically significant. In case of a significant publication bias, the Duval and Tweedie’s trim and fill method was used to allow for the estimation of an adjusted effect size [12].

### Quality assessment

The quality of each included study was assessed. For Randomized Clinical Trial (RCT) it was evaluated according to the Cochrane Collaboration tool for assessing risk of bias: seven distinct domains were identified and evaluated as ‘‘Low risk of bias’’ or ‘‘High risk of bias’’ or ‘‘Unclear’’ (Appendix 1a).

For non-randomized studies, the Newcastle–Ottawa Scale (NOS) was used: the NOS contains eight items, categorized into three domains and a star system is used to allow a semi-quantitative assessment and researchers assign up to a maximum of nine points (Appendix 1b).

## Results

After excluding duplicate results, the search retrieved 196 articles. Of these studies, 101 were excluded because they were off the topic after scanning the title and/or the abstract, 22 because of they were not written in English language, and 3 because no full-text was available. Twenty-four studies were excluded because they were reviews/animal model/case reports and 12 studies were excluded, after full-length paper evaluation, for lack of data. Thus, 34 studies were included in the analysis. [13–46] (Appendix 2).

### Studies characteristics

The included studies comparing the new tracers with the conventional approach to identify the SLN were 34, involving 5882 patients, whereof 3980 cases underwent SLNB with ICG vs conventional tracers [13, 15, 16, 19–21, 25–28, 32, 34–38, 41–46] and 1902 received SPIO vs conventional tracers for SLN identification. [14, 17, 18, 22–24, 29–31, 33, 39, 40]

Major characteristics of included studies are shown in Tables 1 and 2.

**Table 1:** Characteristics of included studies for comparison between SPIO and Conventional Tracers

	Tracer	Number of patients	Mean number of SLN for patient identified	Number of metastatic SLN identified	SLN identification rate
Alvarado et al. [14]	SPIO	146	2.4	24	0.94
	RI + BD	146	2.4	24	0.94
Douek et al. [17]	SPIO	160	2	23	0.94
	RI + BD	160	1.9	24	0.95
Ghilli et al. [18]	SPIO	193	1.8	55	0.98
	RI	193	1.8	56	0.99
Houpeau et al. [22]	SPIO	108	2.01	45	0.97
	RI + BD	108	1.94	44	0.95
Karakatsanis et al. [24]	SPIO	206	1.83	52	0.98
	RI + BD	206	1.79	53	0.97
Karakatsanis et al. [23]	SPIO	183	1.26	24	0.96
	RI	155	1.7	25	0.97
Madrona et al. [30]	SPIO	181	1.63	67	0.91
	RI	181	1.55	69	0.86
Pelc et al. [29]	SPIO	62	2	5	0.92
	RI	62	3	5	0.92
Rubio et al. [31]	SPIO	118	2.21	32	0.98
	RI	118	1.9	32	0.96
Shiozawa et al. [32]	SPIO	30	–	–	0.80
	BD	30	–	–	0.77
Taruno et al. [39]	SPIO	210	–	–	0.95
	RI	210	–	–	0.98
Thill et al. [40]	SPIO	150	1.9	33	0.98
	RI	150	1.8	31	0.97

*SLN* sentinel node, *SPIO* Superparamagnetic iron oxide, *RI* radioisotope, *BD* blue dye

**Table 2:** Characteristics of included studies for comparison between ICG and Conventional Tracers

	Tracer	Number of patients	Mean number of SLN for patient identified	Number of metastatic SLN identified	SLN identification rate
Abe et al. [13]	ICG	128	3.1	19	1.00
	BD	128	1	11	0.68
Agrawal et al. [15]	ICG	103	2.73	28	0.97
	RI + BD	103	3.17	31	0.95
Balladrini et al. [16]	ICG	134			1.00
	RI	134			0.94
Grischke et al. [19]	ICG	105			0.89
	RI	105			0.96
Guo et al. [20]	ICG	198	3	20	0.97
	BD	198	2	4	0.89
Hirano et al. [21]	ICG	108	2.2		0.99
	BD	393	1.6		0.93
Liu et al. [25]	ICG	60	2.95		1.00
	BD	60	1.77		0.88
Mazouni et al. [26]	ICG	122	1		0.82
	RI	122	1		0.97
Ngo et al. [27]	ICG	77			0.96
	RI	77			0.93
Papathemelis et al. [28]	ICG	99		27	0.98
	RI	99		24	0.98
Samorani et al. [32]	ICG	301	2	70	0.99
	RI	301	2	55	0.77
Somashekhar et al. [34]	ICG	100		42	0.96
	RI + BD	100		40	0.94
Sorrentino et al. [35]	ICG	70	1.14	17	0.93
	RI	194	1.01	46	0.95
Sugie et al. [37]	ICG	99	3.4		0.99
	BD	99			0.78
Sugie et al. [36]	ICG	821	2.3	168	0.97
	RI	821	1.7	162	0.97
Tagaya et al. [38]	ICG	25	5.5	8	1.00
	BD	25	2.3	6	0.92
Valente et al. [41]	ICG	92	2.4	24	0.95
	RI	92	2.2	23	0.86
Verbeek et al. [42]	ICG	95		22	1.00
	RI	95		20	0.77
Wang et al. [43]	ICG	70	3.5	18	1.00
	BD	70	2.4	14	0.93
Wishart et al. [44]	ICG	104	1.93	25	1.00
	RI + BD	104		25	0.73
Yuan et al. [45]	ICG	29			0.93
	BD	38			0.90
Zhang et al. [46]	ICG	197	3	51	0.97
	BD	218	2.1	51	0.90

*SLN* sentinel node, *ICG* Indocyanine green, *RI* radioisotope, *BD* blue dye

### SLN identification rate

#### SPIO vs conventional tracers

Twelve studies reported about SLN identification rate with the usage of SPIO vs conventional tracers [14, 17, 18, 22–24, 29–31, 33, 39, 40] and, in details, 8 authors compared SPIO with single tracer (BD or RI) involving 801/1902 patients (42.12%); [18, 23, 29–31, 33, 39, 40] on the other hand, 4 authors reported the comparison between SPIO and dual tracer (BD + RI), involving 1101/1902 patients (57.88%) [14, 17, 22, 24].

**Fig. 1:**
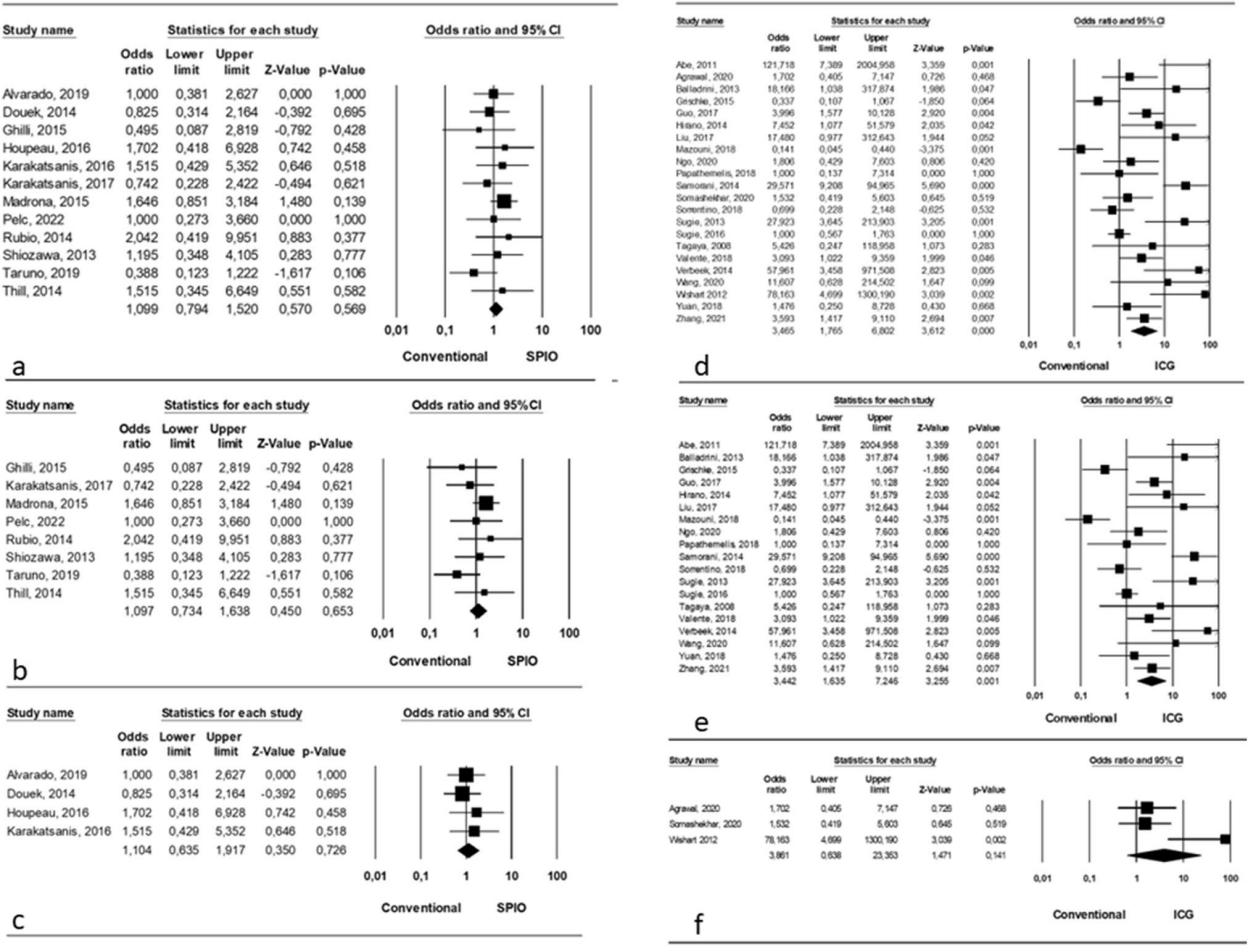
SLN Identification rate **a** Comparison between SPIO and conventional tracers (overall); **b** Comparison between SPIO and conventional single tracer; **c** Comparison between SPIO and conventional dual tracers; **d** Comparison between ICG and conventional tracers (overall); **e** Comparison between ICG and conventional single tracer; **f** Comparison between ICG and conventional dual tracers

Analyzing an overall comparison between SPIO and conventional tracers, we found no statistically significant differences (OR = 1.099, p = 0.569, 95% CI 0.794, 1.520) and no significant heterogeneity among studies (I^2^ = 0.01%; p = 0.74) (Fig. 1a).

Considering the comparison between SPIO and conventional single tracer, we found no significant differences (OR = 1.097, p = 0.653, 95% CI 0.734, 1.638) and no significant heterogeneity among studies (I^2^ = 0.01%; p = 0.46) (Fig. 1b).

Finally, the comparison between SPIO and conventional dual tracer revealed no significant differences (OR = 1.104, p = 0.726, 95% CI 0.635, 1.917) and no significant heterogeneity among studies (I^2^ = 0.01%; p = 0.8) (Fig. 1c).

#### ICG vs conventional tracers

Twenty-two studies reported about SLN identification rate with the usage of ICG vs conventional tracers [13, 15, 16, 19–21, 25–28, 32, 34–38, 41–46] and, in details, 19 authors compared ICG with single tracer (BD or RI) involving 3673/3980 patients (92.28%); [13, 16, 19–21, 25–28, 32, 35–38, 41–43, 45, 46] on the other hand, 3 authors reported the comparison between ICG and dual tracer (BD + RI), involving 307/3980 patients (7.72%) [15, 34, 44].

Analyzing an overall comparison between ICG and conventional tracers, we found a statistically significant difference (OR = 3.456, p = 0.001, 95% CI 1.765, 6.802) in favor of ICG and no significant heterogeneity among studies (I^2^ = 78.87%; p = 0.001) (Fig. 1d).

Considering the comparison between ICG and conventional single tracer, we found a significant difference (OR = 3.442, p = 0.001, 95% CI 1.635, 7.246) in favor of ICG and a significant heterogeneity among studies (I^2^ = 80.57%; p = 0.001) (Fig. 1e).

Finally, the comparison between ICG and conventional dual tracer revealed significant differences (OR = 3.861, p = 0.141, 95% CI 0.638, 23.353) in favor of ICG and a significant heterogeneity among studies (I^2^ = 69.59%; p = 0.03) (Fig. 1f).

### Number of metastatic SLN identified

#### SPIO vs conventional tracers

Ten studies reported the number of metastatic SLN identified with the usage of SPIO vs conventional tracers [14, 17, 18, 22–24, 29–31] for a total of 1662 involved; 6 authors compared SPIO with single tracer (BD or RI) involving 1042/1662 patients (62.69%); [18, 23, 29–31] on the other hand, 4 authors reported the comparison between SPIO and dual tracer (BD + RI), involving 620/1662 patients (37.31%) [14, 17, 22, 24].

**Fig. 2:**
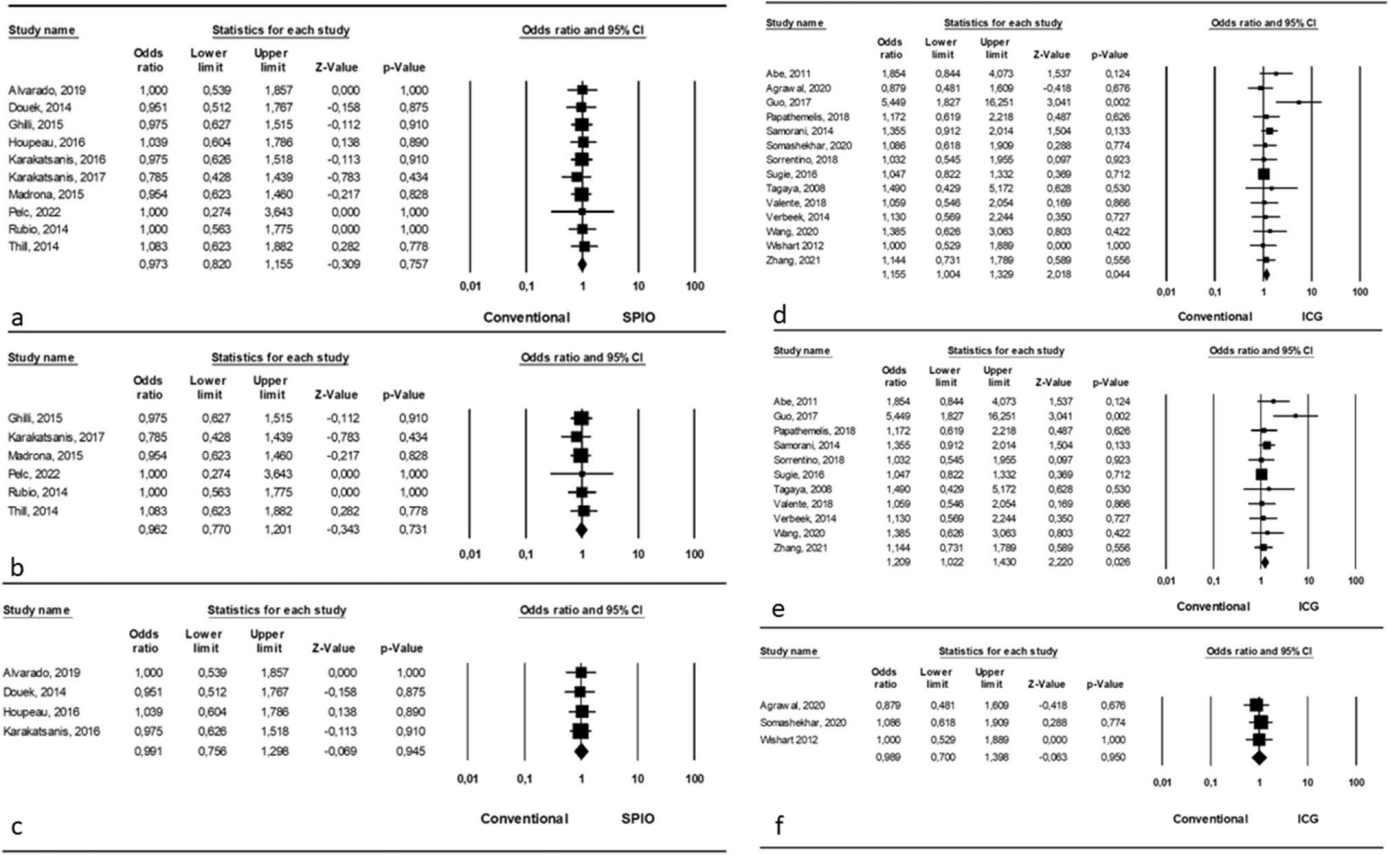
Number of metastatic SLN identified **a** Comparison between SPIO and conventional tracers (overall); **b** Comparison between SPIO and conventional single tracer; **c** Comparison between SPIO and conventional dual tracers; **d** Comparison between ICG and conventional tracers (overall); **e** Comparison between ICG and conventional single tracer; **f** Comparison between ICG and conventional dual tracers

Analyzing an overall comparison between SPIO and conventional tracers, we found no statistically significant differences (OR = 0.973, p = 0.757, 95% CI 0.820, 1.155) with a significant heterogeneity among studies (I^2^ = 0.99%; p = 0.001) (Fig. 2a).

Considering the comparison between SPIO and conventional single tracer, we found no significant differences (OR = 0.962, p = 0.731, 95% CI 0.770, 1.201) and no significant heterogeneity among studies (I^2^ = 0.01%; p = 0.99) (Fig. 2b).

Finally, the comparison between SPIO and conventional dual tracer revealed no significant differences (OR = 0.991, p = 0.945, 95% CI 0.756, 1.298) with a significant heterogeneity among studies (I^2^ = 0.99%; p = 0.001) (Fig. 2c).

#### ICG vs conventional tracers

Fourteen studies reported about the number of metastatic SLN identified with the usage of ICG vs conventional tracers [13, 15, 20, 28, 32, 34, 35, 37, 38, 41–44, 46] and, in details, 11 authors compared ICG with single tracer (BD or RI) involving 2508/2815 patients (89.09%); [13, 20, 28, 32, 35, 37, 38, 41–43, 46] on the other hand, 3 authors reported the comparison between ICG and dual tracer (BD + RI), involving 307/2815 patients (10.91%) [15, 34, 44].

Analyzing an overall comparison between ICG and conventional tracers, we found a statistically significant difference (OR = 1.155, p = 0.04, 95% CI 1.004, 1.329) in favor of ICG and no significant heterogeneity among studies (I^2^ = 0.01%; p = 0.529) (Fig. 2d).

Considering the comparison between ICG and conventional single tracer, we found a significant difference (OR = 1.209, p = 0.02, 95% CI 1.022, 1.430) in favor of ICG and no significant heterogeneity among studies (I^2^ = 7.4%; p = 0.37) (Fig. 2e).

Finally, the comparison between ICG and conventional dual tracer revealed no significant differences (OR = 0.989, p = 0.95, 95% CI 0.700, 1.398) and no significant heterogeneity among studies (I^2^ = 0.01%; p = 0.881) (Fig. 2f).

### Mean number of SLN identified for patient

#### SPIO vs conventional tracers

Ten studies reported the mean number of SLN identified for patient with the usage of SPIO vs conventional tracers [14, 17, 18, 22–24, 29–31, 40] for a total of 1662 involved; 6 authors compared SPIO with single tracer (BD or RI) involving 1042/1662 patients (62.69%); [18, 23, 29–31, 40] conversely, 4 authors reported the comparison between SPIO and dual tracer (BD + RI), involving 620/1662 patients (37.31%) [14, 17, 22, 24].

**Fig. 3:**
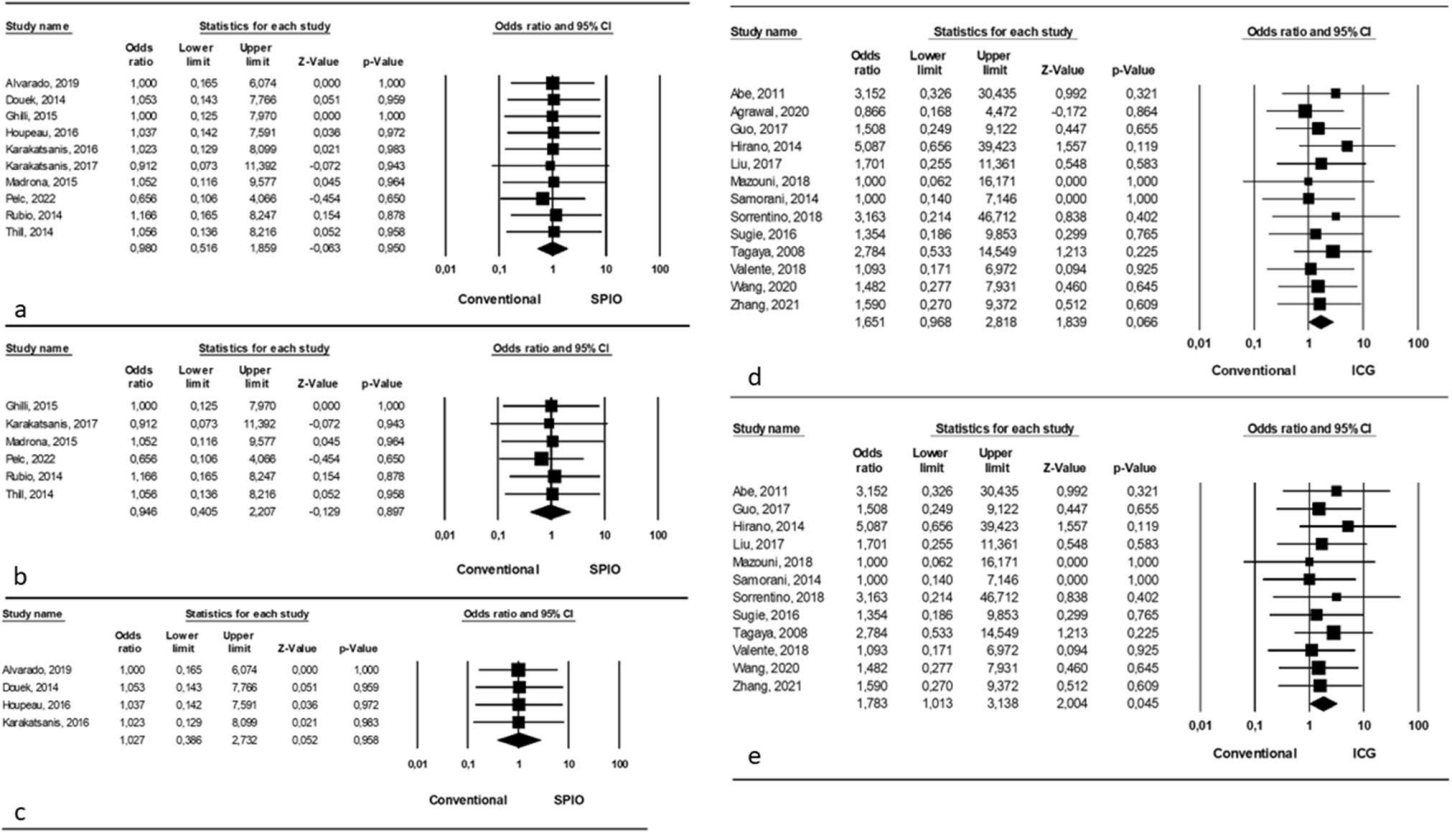
Mean number of SLN identified for patient **a** Comparison between SPIO and conventional tracers (overall); **b** Comparison between SPIO and conventional single tracer; **c** Comparison between SPIO and conventional dual tracers; **d** Comparison between ICG and conventional tracers (overall); **e** Comparison between ICG and conventional single tracer

Analyzing an overall comparison between SPIO and conventional tracers, we found no statistically significant differences (OR = 0.980, p = 0.950, 95% CI 0.516, 1.859) with no significant heterogeneity among studies (I^2^ = 0.01%; p = 0.99) (Fig. 3a).

Considering the comparison between SPIO and conventional single tracer, we found no significant differences (OR = 0.946, p = 0.897, 95% CI 0.405, 2.207) and no significant heterogeneity among studies (I^2^ = 0.01%; p = 0.99) (Fig. 3b).

Similarly, the comparison between SPIO and conventional dual tracer revealed no significant differences (OR = 1.027, p = 0.958, 95% CI 0.386, 2.732) with no significant heterogeneity among studies (I^2^ = 0.01%; p = 0.99) (Fig. 3c).

#### ICG vs conventional tracers

Thirteen studies reported about the mean number of SLN identified for patient with the usage of ICG vs conventional tracers [13, 15, 20, 21, 25, 26, 32, 35, 37, 38, 41, 43, 46] and, in details, 12 authors compared ICG with single tracer (BD or RI) involving 2997/3100 patients (96.67%); [13, 20, 21, 25, 26, 32, 35, 37, 38, 41, 43, 46] on the other hand, just 1 author reported the comparison between ICG and dual tracer (BD + RI), involving 103/3100 patients (3.33%) so no statistical analysis can be performed for this comparison [15].

Analyzing an overall comparison between ICG and conventional tracers, we found no significant differences (OR = 1.651, p = 0.066, 95% CI 0.968, 2.818) and no significant heterogeneity among studies (I^2^ = 0.01%; p = 0.99) (Fig. 3e).

Considering the comparison between ICG and conventional single tracer, we found a significant difference (OR = 1.783, p = 0.04, 95% CI 1.013, 3.138) in favor of ICG and no significant heterogeneity among studies (I^2^ = 0.01%; p = 0.99) (Fig. 3f).

### Publication bias

**Fig. 4:**
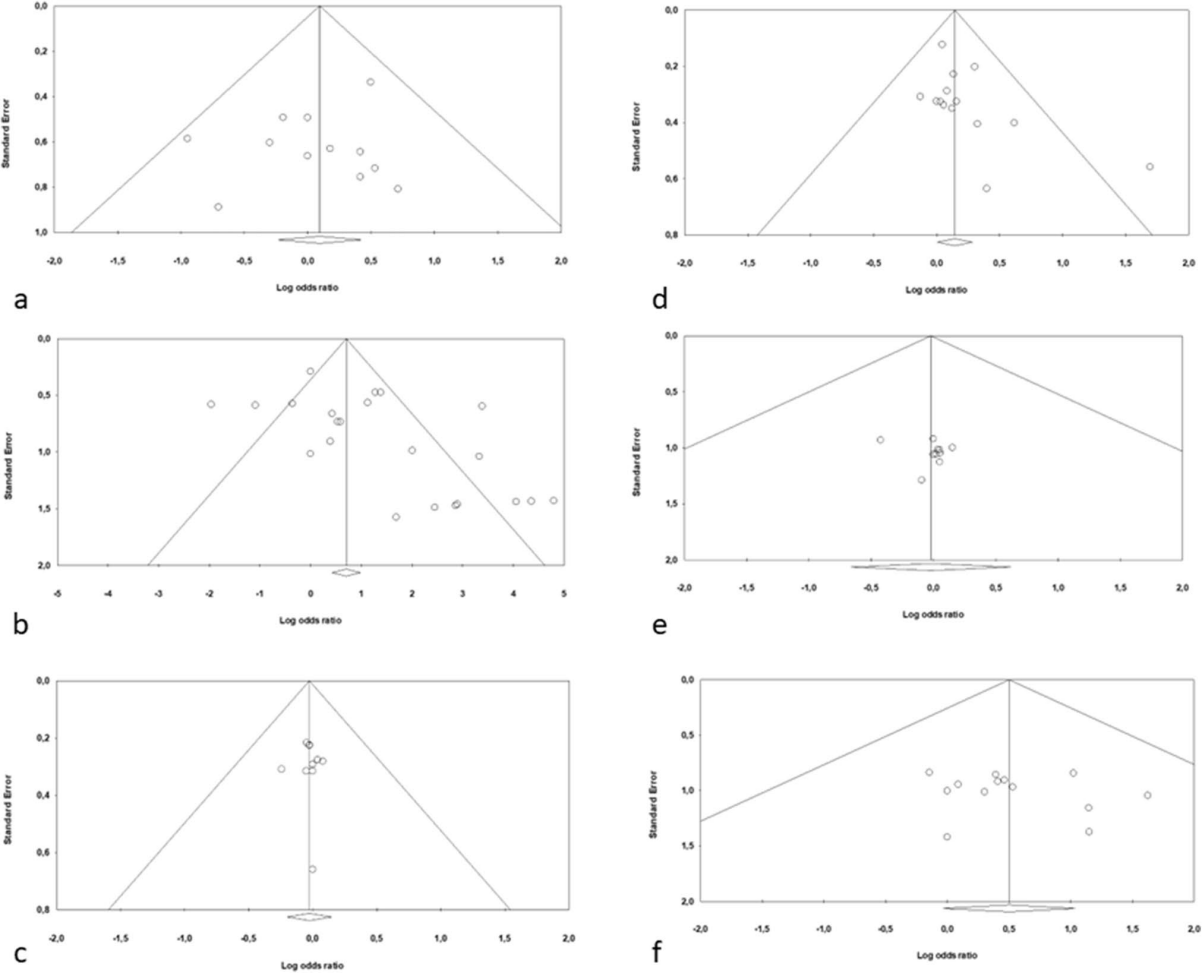
Publication bias **a** Publication bias in the SPIO group for SLN Identification rate; **b** Publication bias in the ICG group for SLN Identification rate; **c** Publication bias in the SPIO group for number of metastatic SLN identified; **d** Publication bias in the ICG group for number of metastatic SLN identified; **e** Publication bias in the SPIO group for mean number of SLN identified for patient; **f** Publication bias in the ICG group for mean number of SLN identified for patient

Because it is recognized that publication bias can affect the results of meta-analyses, we attempted to assess this potential bias using funnel plots analysis. The distribution of studies evaluating SLN identification rate in SPIO group (p = 0.46) was symmetrical and no publication bias was found by the Egger’s test. Conversely, the distribution of studies evaluating SLN identification rate in ICG group highlighted a publication bias at Egger’s test (p = 0.01) (Fig. 4a and b).

About the number of metastatic SLN identified, the distribution of studies of both SPIO group (p = 0.95) and ICG group (p = 0.08) was symmetrical and no publication bias was found by the Egger’s test (Fig. 4c and d).

Finally, about the mean number of SLN for patient identified, the distribution of studies of both SPIO group (p = 0.49) and ICG group (p = 0.43) was symmetrical and no publication bias was found by the Egger’s test (Fig. 4e and f).

### Risk of bias

Apart from Ghilli et al. [18] which realized a good quality RCT, all included studies have a retrospective/prospective design. About these 33 studies [13–17, 19–46], NOS evaluation revealed a 8.12 ± 0.7 mean points with a good quality of evidences.

## Discussion

Our review found no statistically significant differences in terms of SLNs identification rates between SPIO, RI and BD (both as single tracers and as a combined dual technique), while showed significantly higher identification rates with the use of ICG.

No statistically significant differences have been also found for the number of metastatic lymph nodes identified in the comparison between SPIO, RI and BD (both as single tracers and as a combined dual technique). No statistically significant differences have been also reported for the comparison between ICG and conventional tracers used as a dual technique, while statistically significant differences in favor of ICG have been reported for the comparison between ICG and conventional tracers, as overall comparison (i.e. both as dual and single tracers) and as single tracers.

No statistically significant differences in terms of mean number of SLNs identified have been also reported for both the comparison between SPIO and conventional tracers (both as single and dual tracers) and ICG versus conventional tracers.

These results could have a relevant impact on our daily surgical activity, as these new techniques should no longer be considered as investigational.

Particularly, good results in terms of SLNs identification rates and number of metastatic LNs identified have been found for the comparison between ICG and conventional tracers, supporting the use of ICG as a reliable alternative to standard tracers for SLN mapping.

Anyway, some caveat should be taken into account when using ICG for SLN mapping. The technique has not been standardized yet. The needed amount of ICG to be injected actually depends on the surgeon preference that should consider the breast volume and patient’s BMI. The time between the injection and the skin incision should be not shorter than 10 min.

Most of the compared studies use a peri-areola or a non-peritumoral technique; whilst the literature shows that most techniques are equally accurate in identifying the first level 1 axillary node, they are not equivalent in identification rates for additional sentinel lymph nodes which will vary depending on the injection site and technique.

The axillary skin incision should not be made before the tracer has visibly reached the axilla. Otherwise, the lymphatic vessels could be interrupted too soon, making the identification of SLNs more difficult [47]. Moreover, when the first SLN is resected, ICG could leak out, spreading to the surgical field and making it difficult to identify other SLNs.

Furthermore, ICG fluorescence is scattered by superficial tissues, so it could be difficult to be detected in fatty axillas in obese patients [36]. ICG should also not be used in patients with allergy to Iodine [5]. After all, the identification of the sentinel node relies on its visual detection and this limits the identification of sentinel nodes not in the level I of the axilla.

On the other hand, ICG enables a real time visualization of the lymphatic flow from the breast to the axilla, allowing the identification of multiple lymph drainage pathways and multiple SLNs [21]. ICG is also cheaper than radioisotopes, making it possible to perform adequate axillary staging in hospitals where a nuclear medicine service is not available [48].

The good safety profile in terms of reported severe adverse events together with the results of our meta-analysis in terms of accuracy in detecting the SLNs make ICG a reliable alternative to standard methods for SLN mapping, even though the technique should be further refined and standardized to reduce variability among different practices.

The results of our meta-analysis also support the use of SPIO for the identification of SLN in breast cancer, showing non-inferiority of this technique compared to standard tracers in terms of SLN identification rate, number of sentinel lymph nodes identified and number of metastatic lymph nodes identified.

It has to be considered that these new mapping techniques require the passage through a learning curve for breast surgeons: as regards the SPIO, it has been demonstrated that, not differing from the classic approach with RI, a learning curve of about 20 patients is sufficient to perform the technique safely [18]. About ICG, there is no certain data in the literature on the number of procedures necessary for training in axillary SLNB; at same time, according to Khoury-Collado et al., the cut-off for learning curve in SLN biopsy with ICG in endometrial cancer should be fixed at 30 cases [48].

Some limitations are also associated with the use of SPIO [8–10]. For example, the diameter of the magnetometer is larger than the gamma probe and a rebalancing of the probe is required before each signal acquisition. Moreover, there is a possible interference of the surgical instrumentations with the signal. The magnetometer could also show some limits on identifying deeper lymph nodes. Another reported caveat is the persistence of SPIO within the breast tissues, creating potential artifacts in postoperative breast MRI, this limiting the use of this technique for patients undergoing primary systemic therapies or any other patient needing MRI for the post-operative follow-up [9].

Furthermore, this technique could not be used in patients with pacemakers or metal implants or with a known allergy to iron or dextran compounds. Finally, a dermal pigmentation could be reported in up to 20% of patients at the injection site [8–10].

On the other hand, the magnetic tracer could be injected the same day of the surgery directly in the operating room. Moreover, being retained within the SLN, it could allow different useful applications, as the one proposed in the SentiNOT trial for the axillary staging in DCIS patients [49].

These evidence together with the results of our meta-analysis show that SPIO could be considered a safe and reliable alternative to standard tracers for SLN mapping.

The recent network meta-analysis from Mok et al. comparing ICG, SPIO, Tc and blue dye found pooled risk ratios of Tc, ICG and SPIO showed statistically better performance in detecting sentinel lymph nodes than blue dye alone. ICG had the lowest false-negative rate, followed by Tc and SPIO, with blue dye alone as the reference group; authors concluded that SPIO or ICG alone are superior to blue dye alone and comparable to the standard dual-modality technique of blue dye with Tc. [50]

Liu et al. perform a systematic review and meta-analysis to evaluate the diagnostic accuracy of SPIO and its clinical impact: they concluded that SPIO could be considered as an alternative standard of care for sentinel lymph node detection, with an equivalent or even superior detection capacities compared with standard techniques [51]. Another review was conducted by Ferrucci et al. who concluded that the new SLNB techniques seem to be safe, feasible and have shown very high improvements in accuracy, sensitivity and specificity in last years; all the last evidences show similar results or better than the traditional approaches and made surgeon independent from the nuclear medicine department [4].

Similar results were reached by Bove et al., who realized a narrative review underlining that the contrast-enhanced ultrasound (CEUS) is an active field of research but cannot be recommended for clinical use at this time. The ICG fluorescence technique was superior in terms of detection rate, as well as having the lowest false negative rate. The detection rate descending order was SPIO, Tc, dual modality (Tc/BD), CEUS and BD [52].

Also Goyal conducted a review on the novel techniques for sentinel node detection: he concluded that the newer developing techniques will potentially enable a more widespread adoption, and for many sites with no access to radioisotope Sienna + or ICG are being used routinely; he also underlined that CEUS has the potential to improve the sensitivity of conventional grey-scale US and stage the axilla non-operatively [53].

Niebling and colleagues conducted a systematic review of the literature on SLNB in patients with early stage breast carcinoma and melanoma, pooling data from 158 studies and 44,172 patients: they found SLN identification using solely blue dye was 85% and 84%, while for radiocolloid alone it was 94% and 99%, respectively. Using a combination of radiocolloid and blue, identification rates were 95% and 98% [54].

It could be also interesting to evaluate the costs of these new techniques in SLN identification: Shams and colleagues performed a cost-analysis of Magtrace^®^ compared with standard Tc and found that Magtrace localization shortened the preoperative care pathway and did not affect surgical time or economical reimbursement [55]. Similarly, Khadka et al. designed an RCT to compare fluorescein + methylene blue and Tc-99 m sulfur colloid + methylene blue in sentinel node biopsy: the trial demonstrated noninferiority of fluorescein + methylene blue and they found the fluorescein + methylene blue was more cost-effective than isotope guided sentinel node biopsy [56].

These papers state that the new identification techniques, in addition to being effective and safe, also have a good cost-effectiveness ratio.

This meta-analysis has some limitations, mainly due to the significant heterogeneity found among included studies for most comparisons.

It’s also important to highlight that this systematic review refers to axillary nodes only and the majority of comparative studies do not evaluate extra-axillary nodes or even infraclavicular (level II/III sentinel nodes).

Moreover, the quantification of mean number of sentinel nodes harvested is a problematic measurement as most studies fail to specify how additional sentinel nodes are identified (adjacent nodes taking up tracer or having activity in excess of surrounding non sentinel nodes). Finally, it has to be considered that, apart from Ghilli et al. [18], there is a lack of RCT and this exposes our analysis to major risks of bias although NOS assessment revealed a good quality of evidences.

In conclusion, results obtained from the analysis of available studies comparing SPIO and ICG with standard tracers offer reliable evidence supporting the use of both ICG and SPIO for the pre-operative mapping of sentinel lymph nodes in breast cancer treatment.

## Supplementary Information

Below is the link to the electronic supplementary material.

Supplementary file1 Appendix 1a: Cochrane Collaboration tool for assessing risk of bias (JPG 47 KB)

Supplementary file2 Appendix 1b: NOS Quality assessment (JPG 55 KB)

Supplementary file3 Appendix 2: PRISMA flowchart (JPG 302 KB)

## Data Availability

Not applicable.
