# Supplementary figures and images for: New techniques versus standard mapping for sentinel lymph node biopsy in breast cancer: a systematic review and meta-analysis
Source: Updates Surg. 2023 Jun 16;75(6):1699–710. doi: 10.1007/s13304-023-01560-1 (PMC10435404; doi:10.1007/s13304-023-01560-1)

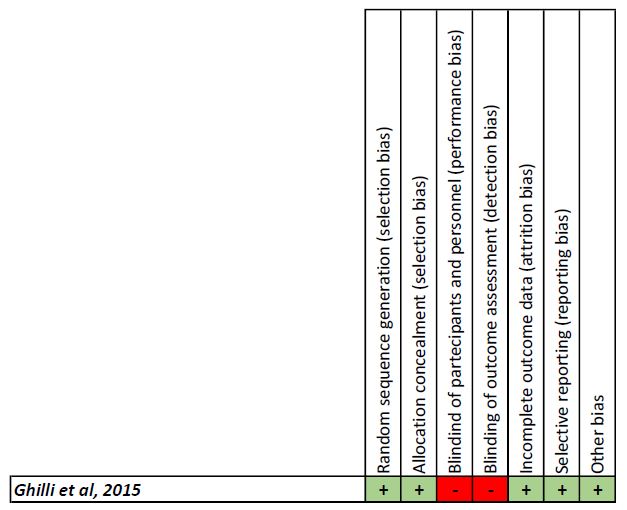

Supplement: Supplementary file 1 — Supplementary file1 Appendix 1a: Cochrane Collaboration tool for assessing risk of bias (JPG 47 KB) [file 13304_2023_1560_MOESM1_ESM.jpg]

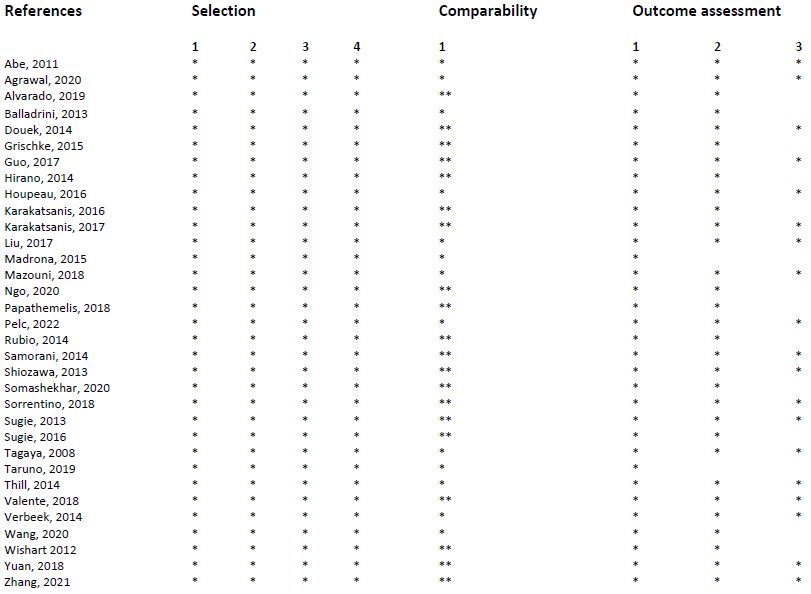

Supplement: Supplementary file 2 — Supplementary file2 Appendix 1b: NOS Quality assessment (JPG 55 KB) [file 13304_2023_1560_MOESM2_ESM.jpg]

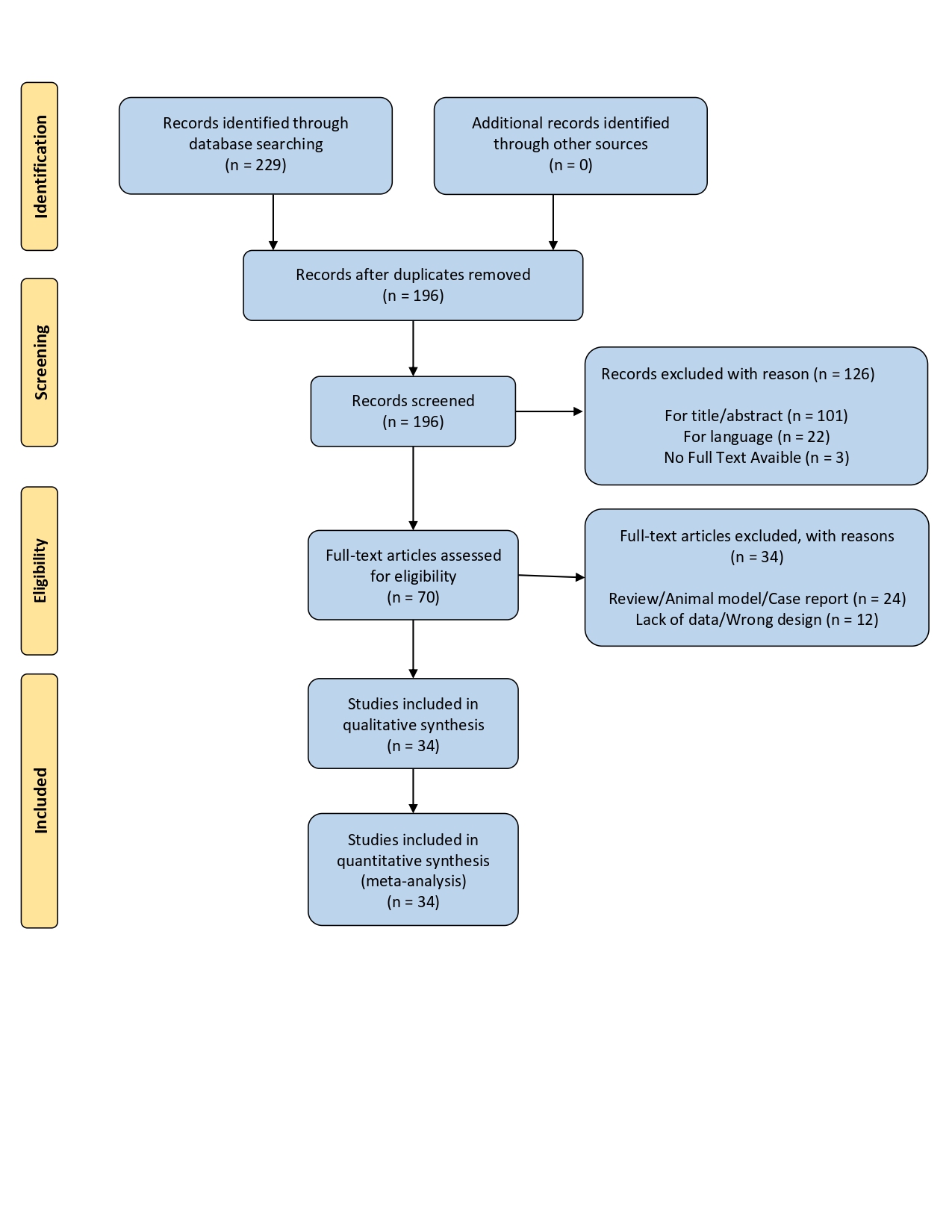

Supplement: Supplementary file 3 — Supplementary file3 Appendix 2: PRISMA flowchart (JPG 302 KB) [file 13304_2023_1560_MOESM3_ESM.jpg]
